# Editorial: Insights in multiple sclerosis and neuroimmunology: 2021

**DOI:** 10.3389/fneur.2023.1251877

**Published:** 2023-07-20

**Authors:** Robert Weissert

**Affiliations:** Department of Neurology, University of Regensburg, Regensburg, Germany

**Keywords:** autoimmunity, central nervous system, multiple sclerosis, autoimmune encephalitis, biomarkers, clinical outcomes

A total of 19 articles are published in the Frontiers Research Topic “*Insights in multiple sclerosis and neuroimmunology 2021*.” These cover a wide aspect of multiple sclerosis (MS)- related themes as well as themes in autoimmune encephalitis.

There is a need for consensus criteria about the identification of certain types of MS. Meca-Lallana et al. show in their article “*Consensus on early detection of disease progression in patients with multiple sclerosis*” that such consensus statements could help clinicians to find early in the disease course patients with secondary progressive MS (SPMS). Such an early identification is important to perform adequate therapeutic management. Standardized clinical assessments are meaningful in MS care. In the article “*Making every step count: minute-by-minute characterization of step counts augments remote activity monitoring in people with multiple sclerosis*,” Block et al. used a model to predict disease progression over the longer term (>2 years) based on obtained measurements. These findings will be used to develop further descriptive metrics for activity. In their article “*Current status and future opportunities in modeling clinical characteristics of multiple sclerosis*,” Liu et al. suggest that there is a strong need to develop validated models of MS clinical outcomes by using cellular or/and molecular biomarkers. In the article titled “*Models of care in multiple sclerosis: a survey of Canadian health providers*,” Marrie, Donkers et al. claim that the ideal MS service is multidisciplinary in nature, ideally integrated, and with prompt access to care.

The myxovirus resistance protein A (MxA) has been long used as a marker for exogenous interferon-beta efficacy in MS treatment. Coerver et al. show in the article “*The association between blood MxA mRNA and long-term disease activity in early multiple sclerosis*” that MxA mRNA is expressed in inflammatory pathology in MS that is dependent on the endogenous type-1 interferon system and that this might be a prognostic biomarker for long-term inflammatory disease activity in MS. In the article “*Genetic risk variants for multiple sclerosis are linked to differences in alternative pre-mRNA splicing*” by Putscher et al., the authors show that genetic variants from MS risk loci affect pre-mRNA splicing. Amoriello et al. investigated soluble HLA-G (sHLA-G) levels in MS in the article “*Investigating serum sHLA-G cooperation with MRI activity and disease-modifying treatment outcome in relapsing-remitting multiple sclerosis*.” They found that the HLA-G genotype strongly influences sHLA-G levels. Autoantibodies are of importance in various neuroimmunological disorders, and their role and mechanism of action are partly undefined. In the article “*Peptidylarginine deiminase 2 autoantibodies are linked to less severe disease in multiple sclerosis and post-treatment Lyme disease*,” Kim et al. make the case that anti-peptidylarginine deiminase 2 (PAD2) antibodies may attenuate inflammation. This effect is observable in tissues with high expression of PAD2. The role of hemolysis was analyzed in the article “*Peripheral hemolysis in relation to iron rim presence and brain volume in multiple sclerosis*” by Krajnc et al. The authors found an influence of hemolysis on the brain volume but not on the presence of iron rim lesions in progressive MS. Investigations about metabolomics in neuroimmunological disorders is of increasing interest. In their study “*Metabolomics of cerebrospinal fluid in multiple sclerosis compared with healthy controls: a pilot study*,” Židó et al. investigated cerebrospinal fluid (CSF) from MS patients compared to controls regarding metabolomic profiles. They found differences in amino and fatty acids in the CSF of newly diagnosed patients with MS in comparison with controls. The most significant changes were seen in levels of arginine, histidine, and palmitic acid. They concluded that such a metabolomic profile may predict inflammatory disease activity in MS. In the article “*Effects of vascular comorbidity on cognition in multiple sclerosis are partially mediated by changes in brain structure*,” Marrie, Patel et al. showed that vascular comorbidity leads to changes in brain macrostructure and microstructure. In addition, this is associated with lower cognitive function in patients with MS.

In “*Bridging therapies with injectable immunomodulatory drugs in the management of multiple sclerosis: a Delphi survey of an Italian expert panel of neurologists*,” Marfia et al. suggest that the value of bridging therapy with injectable immunomodulatory drugs in MS disease conditions is underscored. The article focuses on patients with MS who plan to become pregnant and patients with MS at risk for cancer recurrence. Ozanimod is a selective sphingosine-1-phosphate (S1P)-receptor 1 (S1P1) and S1P5 modulator used for the treatment of active forms of relapsing-remitting MS (RRMS). Ziemssen et al. present their real-world and long-term study “*OzEAN study to collect real-world evidence of persistent use, effectiveness, and safety of ozanimod over 5 years in patients with relapsing-remitting multiple sclerosis in Germany*.” The results of this study will add to the safety profile and efficacy profile of ozanimod in the treatment of RRMS. In the study “*Safety, adherence and persistence in a real-world cohort of German MS patients newly treated with ocrelizumab: first insights from the CONFIDENCE study*,” Weber et al. describe the safety profile of ocrelizumab in the CONFIDENCE real-world MS population study. The findings were consistent with the findings in pivotal clinical trials for the anti-CD20 B cell-depleting antibody ocrelizumab used for the treatment of patients with RRMS and patients with primary progressive MS (PPMS). Importantly, high treatment persistence and adherence were seen in this real-world MS population study. Fathi et al. suggested in their article, “*Dynamic changes in kynurenine pathway metabolites in multiple sclerosis: a systematic review*,” that quinolinic acid is a possible player in the pathogenesis of MS. This conclusion is mainly based on the finding that quinolinic acid levels in CSF were higher in patients with MS than in healthy controls. The value of disease models induced in mice and rats on certain novel MS therapeutic approaches is outlined by Jayaraman and Jayaraman in their article “*Impact of histone modifier-induced protection against autoimmune encephalomyelitis on multiple sclerosis treatment*” about histone deacetylase (HDAC) inhibitors. HDAC inhibitors such as valproic acid and hydroxamates as well as others are possible candidates for future treatment of MS.

It has been shown that in paraneoplastic forms of autoimmune encephalitis, the removal of the associated cancer entity is of primary importance in long-term disease outcomes. For teratoma, Zhang et al. show in their article “*Long-term prognosis of patients with anti-N-methyl-D-aspartate receptor encephalitis who underwent teratoma removal: an observational study*” that early detection and removal of teratoma resulted in a favorable long-term prognosis in patients with anti-NMDAR encephalitis. Case studies can be of importance for defining potential new disease entities and for the description of rare disease variants. In the case study “*Acute cerebellitis associated with anti-homer 3 antibodies: a rare case report and literature review*” by Miao et al., the authors underscore the need for immune-mediated causes to be considered in acute cerebellitis. Importantly, immunotherapy can contribute to the improvement of cerebellar syndrome. Neuropsychological assessment is important in phenotyping and care of patients with neuroimmunological disorders and especially autoimmune encephalitis. In the article by Chan et al., “*Cognitive and mood profiles among patients with stiff person syndrome spectrum disorders*,” it is clarified that neuropsychological testing in stiff person syndrome should include testing of verbal learning and recall, phonemic verbal fluency, attention, and processing speed.

In conclusion, the Research Topic “*Insights in multiple sclerosis and neuroimmunology 2021*” gives novel insight into current research themes on MS and autoimmune encephalitis.

## Author contributions

RW outlined and wrote the Editorial.

